# Advancing healthcare AI governance through a comprehensive maturity model based on systematic review

**DOI:** 10.1038/s41746-026-02418-7

**Published:** 2026-02-11

**Authors:** Rowan Hussein, Anna Zink, Bashar Ramadan, Frederick M. Howard, Maia Hightower, Sachin Shah, Brett K. Beaulieu-Jones

**Affiliations:** 1 Center for Computational Medicine and Clinical Artificial Intelligence, Department of Medicine, University of Chicago, Chicago, IL USA; 2 Center for Applied AI, Booth School of Business, University of Chicago, Chicago, IL USA; 3 Equality AI, Park City, UT USA

**Keywords:** Machine learning, Health care

## Abstract

Artificial Intelligence (AI) deployment in healthcare is accelerating, yet governance frameworks remain fragmented and often assume extensive resources. Through a systematic review of 35 frameworks for AI implementation in healthcare (published 2019–2024), we identified seven critical domains of healthcare AI governance. While existing frameworks provide valuable guidance, the resource requirements create barriers for smaller healthcare organizations. To address this gap, we organized key findings from the review to create the Healthcare AI Governance Readiness Assessment (HAIRA), a five-level maturity model that provides actionable governance pathways based on organizational resources. HAIRA spans from Level 1 (Initial/Ad Hoc) to Level 5 (Leading), with specific benchmarks across all seven governance domains. This tiered approach enables healthcare organizations to assess their current AI governance capabilities and establish appropriate advancement targets. Our framework addresses a critical need for adaptive governance strategies that ensure that AI implementation delivers tangible benefits to systems of varying resource levels.

## Introduction

Artificial Intelligence (AI) is being rapidly deployed in many aspects of healthcare, with both predictive and generative AI applications poised for continued expansion^1^. These technologies show considerable promise in addressing critical healthcare challenges, such as offering clinical decision support based on predictive models of patient risk or streamlining administrative tasks for physicians^2,3^. However, as AI/ML tools become more ubiquitous, it is crucial to focus on how they are governed: how they are developed or selected, how they are deployed and integrated into clinical workflows, and how their performance and impact on endpoints like patient care, health equity, and organizational efficiency are measured and monitored. A substantial proportion of this governance may be dictated by legal frameworks overseeing medical devices. The U.S. Food and Drug Administration has proposed a regulatory framework for AI/ML software serving as a medical device^4^, and the European Union (EU) AI Act^5^ creates similar safeguards for applications for EU member states. Successful governance of new technologies in healthcare requires a structured approach to manage their implementation as well as to foresee, measure, and mitigate the consequences of the new technology. This is particularly crucial in medical settings, where suboptimal performance or unexpected outcomes can have life-altering implications. For instance, inadequately governed AI tools may inadvertently perpetuate systemic inequities, as evidenced by algorithms that unintentionally deprioritize care for underserved populations^6^. Effective governance is essential to ensure that technological advancements in healthcare are ethically implemented, efficiently utilized, and consistently prioritize patient welfare.

As AI solutions have emerged, a wide variety of governance best practices in the form of frameworks, governance structures, checklists, and guidelines have been proposed^7–23^. Many focus on methods for assessing the clinical need for AI tools, algorithm development, implementation, and maintenance. However, there remains sparsity around recommendations for organizational structures, external algorithm or model evaluation, and the effects of AI implementation on the downstream outcomes that are critical as opposed to intermediate metrics such as model accuracy. Some frameworks or aspects of frameworks are not feasible for many health systems due to resource and expertise constraints. Additionally, there is substantial variation between published recommendations, and it can be difficult to decide which approach fits each institution. These limitations illustrate the need for cohesive and comprehensive guidance to ensure responsible, successful, and effective AI/ML applications in healthcare. Strong AI governance does not need to slow down the adoption of AI but should instead provide pathways to systematically identify and mitigate risks and weaknesses, accelerate the adoption among clinicians or other end users, compare between AI-based vendors, products, or models, deploy and integrate into workflows, measure its impact, and monitor for future performance degradation.

Despite extensive discussions on AI led by researchers and engineers, there remains a critical gap in addressing the needs and perspectives of clinicians in the clinical setting. While the broader literature on AI implementation spans diverse topics—including organizational frameworks—healthcare environments present unique challenges such as patient safety, ethical considerations, and workflow integration, all of which directly impact clinical decision-making and patient outcomes. Tailoring AI implementation guidance specifically for clinical settings is essential, as they are ultimately responsible for patient care and must be equipped to critically evaluate and adopt AI tools in practice.

The objective of this systematic review is to systematically map the current landscape of comprehensive guidance for AI implementation in clinical settings, with a focus on how evaluation frameworks are designed and applied. By synthesizing available evidence, we aim to highlight best practices, identify persistent gaps, and propose recommendations that empower clinical settings to lead and shape the responsible integration of AI in healthcare. We evaluated these documents based on their outlined recommendations across key domains of AI governance: organizational structure, problem formulation, external algorithm evaluation and selection, algorithm development and training, model evaluation and validation, deployment and integration, and monitoring and maintenance. In synthesizing the overarching recommendations, we aimed to clarify any existing gaps and provide an understanding of the resources necessary to successfully follow these recommendations. Existing proposed frameworks tended to be geared towards large academic health systems with substantial resources and personnel with expertise in many areas, including but not limited to AI, data science, statistics, implementation science, healthcare IT and EMR integration, business intelligence and reporting, and quality improvement. There are a few actionable pathways tailored to healthcare organizations with varying levels of resources and expertise. As a result, we propose a readiness evaluation framework entitled “Healthcare AI governance readiness assessment” (HAIRA).

This framework aims to provide a method for healthcare systems to assess their current AI governance and to establish appropriate targets for the future. The introduction of a tiered evaluation system is consistent with observations from current leaders in the field. Interviews conducted within academic medical centers (AMCs) have identified three existing governance phenotypes for AI evaluation: well-defined, emerging, and interpersonal, each shaped by the available resources and infrastructure at individual institutions^24^. Furthermore, stakeholders have underscored that, even at this early stage of AI implementation, inadequately evaluated models may present risks to patients^24^. There is a pressing need for robust AI evaluation methodologies, particularly given that while the majority of US hospitals employ predictive models, only half assess these systems for bias and two-thirds for accuracy. These findings highlight a substantial gap in current practices, emphasizing the critical importance of advancing evaluation frameworks as AI becomes increasingly integrated into clinical care.

## Results

**Fig. 1:**
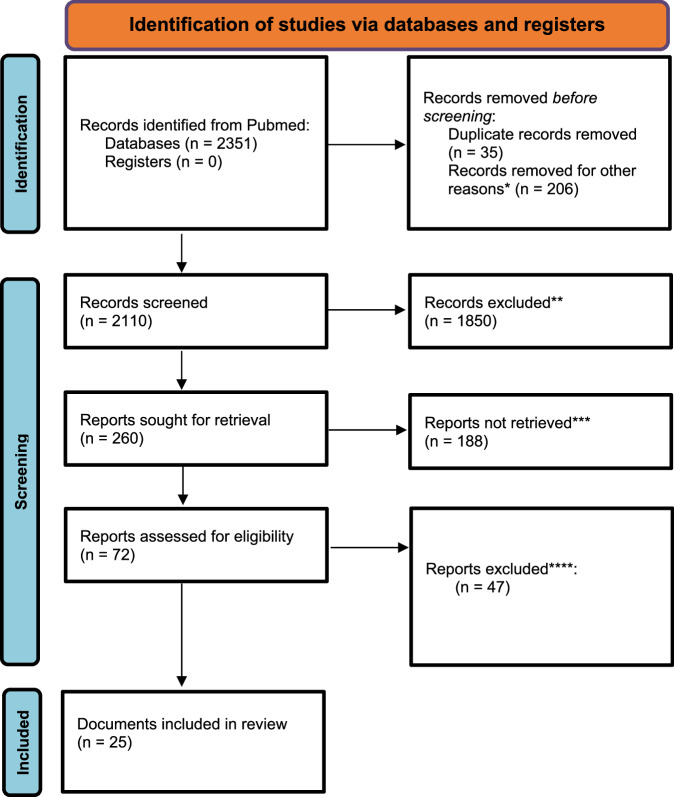
Literature identification and filtering process (PRISMA 2020 flow diagram). The diagram delineates our systematic process for document identification and selection. Our initial search strategy focused on frameworks, governance structures, and checklists about artificial intelligence (AI) in healthcare. After a comprehensive review, articles were excluded if they did not present clear guidelines or if they focused exclusively on a single AI model or a specific aspect of AI implementation. *Limited search to 2019–2024 **Documents were excluded if they did not provide an assessment mechanism for implementation, either via guidelines, checklist or framework. Any papers not written in English were excluded. *** Document that presented guidelines for the implementation of tools that were not AI-based was removed. ****Documents were excluded if no clear assessment or criteria for evaluation were proposed, and simply presented general considerations. Reviews that only analyzed existing frameworks without presenting their own frameworks were excluded. Frameworks that only focused on 1 aspect of implementation, i.e., ethics, were not included as this review focused on a comprehensive tool.

Initially, the search of keywords generated 2351 articles. Applying a temporal filter to focus on recent developments (2019–2024) and removing duplicates reduced this to 2110 articles. Further refinement by limiting the search to carefully selected relevant journals resulted in 260 articles. These articles were manually reviewed to identify those articles addressing or proposing structures for AI governance in health care. The search process resulted in a final inclusion of 29 articles, including 2 additional articles from grey literature and 2 articles not indexed in the database, to ensure in-depth analysis (Fig. 1).

We reviewed and identified recommendations from the 29 selected articles. Recommendations were organized into the following seven categories based on widely recognized processes involved in the development and implementation of AI governance^25–27^ (Table 1).

**Table 1:** High-level categorization of recommendations common to the identified articles

Category	Description
1. Organizational structure	Leadership and teams responsible for AI tool selection and evaluation
2. Problem formulation	Assessment of the clinical issue addressed by the AI tool, including input and output specifications
3. Algorithm development and model training	Application development, including algorithm design and data collection/dataset selection
4. External product evaluation and selection	Pre-launch testing by external parties to assess generalizability beyond the training population
5. Model evaluation and validation	Evaluation by healthcare systems to ensure applicability to specific populations and assess potential risks, errors, or gaps
6. Deployment and integration	Implementation of the software/tool into healthcare system workflows
7. Monitoring and maintenance.	Post-launch assessments to evaluate tool reliability and success as defined by initial problem formulation

The selected articles and their recommendation categories are presented in Table 2. Across the literature, emphasis is placed on problem formulation, algorithm development/model training, and monitoring/maintenance. Conversely, organizational structure and external algorithm evaluation received less attention. Where helpful, we cite single-aspect exemplars (e.g., detailed monitoring or deployment change-management playbooks) to operationalize specific steps, while keeping the evidence base centered on multi-domain governance frameworks.

**Table 2:** Compilation of documents with checklists, guidelines, and frameworks with a primary focus on the regulation of Artificial Intelligence (AI) implementation in the healthcare industry

Paper	Organizational structure	Problem formulation	External algorithm evaluation and selection	Algorithm development and model training	Model evaluation and validation	Deployment and integration	Monitoring and maintenance	Total categories covered
Abràmoff et al.^7^		✓	✓	✓	✓	✓	✓	6
Bedoya et al.^8^	✓	✓	✓	✓	✓	✓	✓	7
van de Sande et al.^49^		✓	✓	✓	✓		✓	5
Callahan et al.^48^	✓	✓		✓	✓	✓	✓	6
CHAI (grey)^28^	✓	✓		✓	✓	✓	✓	6
Chen et al.^9^	✓	✓					✓	3
Chin et al.^10^	✓			✓				2
Coombs et al.^11^		✓			✓			2
Dagan et al.^29^	✓	✓	✓	✓	✓	✓	✓	7
Finlayson et al.^15^							✓	1
Gallifant et al.^50^		✓	✓	✓	✓			4
Hassan et al.^51^	✓			✓	✓	✓	✓	5
de Hond et al.^14^		✓	✓	✓	✓	✓		5
Kashyap et al.^52^	✓							1
Khan et al.^53^	✓	✓		✓		✓	✓	5
Kim et al.^30^	✓	✓		✓	✓	✓	✓	6
Liao et al.^54^		✓			✓		✓	3
NIST (grey)^31^	✓	✓			✓	✓	✓	5
Nyariro et al.^16^		✓						1
Obermeyer et al.^6^	✓			✓			✓	3
Price et al.^41^	✓							1
Reddy et al.^17^	✓					✓		2
Roski et al.^18^		✓	✓	✓	✓	✓	✓	6
Saenz et al.^55^	✓				✓	✓	✓	4
Subbaswamy et al.^19^				✓	✓			2
van der Vegt et al.^20^						✓		1
Wang et al.^21^		✓		✓		✓		3
Whittaker et al.^22^	✓				✓	✓		3
Wiens et al.^23^	✓			✓			✓	3
Total number of papers addressing category	17	16	6	16	16	16	17	

Each document was reviewed, and recommendations were mapped to a specific domain of AI governance.

Seven articles encompassed at least six of the seven domains of AI governance. The insights and recommendations from these comprehensive works have been analyzed and incorporated into our broader synthesis and generalizations. Below is a broad summary of their foci:

Abramoff et al.^7^ developed a framework focused on promoting health equity and bias throughout the AI lifecycle. The framework is not intended to be a comprehensive strategy for mitigating all bias and risks, but it attempts to highlight critical areas of evaluation to ensure overall improvements. Many of the sentiments are echoed throughout the recommendations in the other literature.

Bedoya et al.^8^, often referenced as ABCDS (Algorithm-Based CDS), created a governance framework built around the AI/ML development and implementation process with several checkpoints, which act as software regulatory stop points. This framework has already been deployed in a healthcare system, the Duke University Health System, and at the time of publication, 52 models were regulated via the framework.

CHAI AI governance^28^ (grey literature) was developed with The Joint Commission and emphasizes risk-based management, multidisciplinary governance teams, and broad-level accountability to ensure AI use aligns with ethical standards and regulatory compliance. The framework also incorporates data protection, transparency with patients, and continuous monitoring to address bias and improve outcomes.

Dagan et al.^29^ developed the OPTICA tool, which emerged as the most comprehensive of all the frameworks evaluated and heavily touched, all with great detail, every aspect of the AI life cycle.

Kim et al.^30^, HEAAL framework, is a comprehensive tool that evaluates AI across several domains: accountability, fairness, fitness to purpose, reliability and validity, and transparency throughout the lifecycle.

NIST AI Risk Management Framework (AI RMF)^31^ (grey literature) is built around four iterative, interconnected functions: govern, map, measure, and manage, with the objective of enabling trustworthy AI by emphasizing transparency, fairness, accountability, and robustness.

Finally, Roski et al.^18^ reviewed published literature to evaluate potential areas of risk around each phase of AI development and implementation and designed evidence-based practices for mitigation.

As seen across comprehensive frameworks, a major question remains around the feasibility of all health systems to apply these steps.

### Review of governance recommendations

Table 3 consolidates key recommendations from existing frameworks, including those supported by at least two reviewed articles. By emphasizing recurring themes, it offers an initial framework for tackling governance challenges across the AI lifecycle.

**Table 3:** Harmonized high-level recommendations based on inclusion in multiple published frameworks

Phase	Recommendations
Organizational structure	• Create a governance committee consisting of diverse stakeholders, including data scientists/ML learning experts, AI developers, clinical experts, ethical and legal experts, and patient/target group. • Stakeholders should be involved in all aspects of AI governance, including the deployment, monitoring, and general evaluation.
Problem formulation	• Investigate the current standards of care to gain an understanding of the status quo, workflow, and context to develop a clear and objective rationale for the use of AI. • Outline specific objectives and outcomes for AI tools to achieve and articulate the motivation for choosing a specific model.
External algorithm evaluation	• Governance panel or hospital system should evaluate data utilized for training to ensure it is representative and there is a clear rationale. Conduct validation analysis to evaluate the overall generalizability of data.
Algorithm development and model training	• AI development team should confirm that the data are compliant with relevant privacy legislation and has taken measures to ensure privacy is maintained in the deployment and integration of the tool. • Thorough assessment of collected data to confirm it is representative of the target population and aligned with the intended use, setting, and other relevant characteristics. • Final structure should be well described, and a committee should thoroughly evaluate how the tool will be implemented with a focus on workflow and UI design.
Model evaluation and validation	• Ensure the training data reflect the target population via internal retrospective evaluation • Internal validation to quantify optimism and overfitting. • External validation to assess generalizability. • Prospective analysis through silent monitoring.
Deployment and integration	• Educated end users on the objectives of the tool as well as expected outcomes, potential biases, legal framework, and other benefits and limitations. Evaluate for human biases as well. • Create a system to identify potential sources of risks and where individuals can report errors, failures, and misses. • Shadow deployment to assess for risks, errors, and biases ahead of full integration.
Monitoring and maintenance	• A real-time system should be in place to log, audit, and test bias, accuracy, predictability, and transparency of decision-making. • Monitor for dataset shifts and feedback loops. • Updates and adaptations should occur, but cautiously, and there should be measures to abort should there be major errors.

### Organizational structure

A recurring theme across multiple articles was the necessity for an overarching governance body^7–10,17^. This entity would not only assist in selecting AI tools and software for implementation but also guide the validation, implementation, and monitoring processes. The literature generally recommended that this body comprise a diverse set of stakeholders, including data scientists and machine learning experts, AI developers versed in the technical aspects of the tools, clinical experts to provide insights on output accuracy and field-specific needs, ethical and legal experts, and representatives from patient and target groups. Emphasis was placed on integrating these experts throughout all phases of the AI lifecycle to ensure both efficacy and responsible implementation. Similar to governance of traditional clinical decision support tools, a larger governance structure will be needed for custom, i.e., internally developed AI tools, as opposed to vendor, i.e., commercially developed solutions.

Chen et al. and Reddy et al. proposed a structure of subpanels, overseen by an executive-level overarching committee, each with specific foci to spearhead different aspects of evaluation throughout the AI lifecycle^8,17^. For example, the monitoring phase could be led by a committee with IT expertise to ensure seamless integration of the model into clinical workflows and to evaluate potential errors^8^. This multi-tiered approach aims to provide comprehensive oversight while allowing for specialized attention to each critical phase of AI implementation in healthcare settings.

### Problem formulation

Most articles briefly addressed the importance of clear objectives for AI implementation in healthcare, with only a few offering actionable guidance^9,11,14,17,21^. Healthcare systems were advised to evaluate current standards of care to understand existing workflows. By contextualizing the status quo, health systems are able to rationalize the need for specific AI tools and choose tools that align with population needs.

A subset of articles provided more detailed guidance. For instance, after identifying an aim, de Hond et al. suggested outlining clinical success criteria, for example, specific patient outcomes, and assessing potential risks, including prediction errors^14^. This comprehensive approach aims to ensure that AI tools meet specific healthcare needs and align with safety and efficacy standards in clinical practice.

### Algorithm development and model training

Guidelines specific to algorithm development are crucial for ethical and compliant use of AI tools. They help to mitigate risk such as bias, privacy breaches and potential harm to patients while maintaining public trust in AI technology. An example denoted by Obermeyer et al. is that of an AI algorithm which utilized health care costs a proxy to health need leading to a false conclusion that Black patients were healthier than their white counterparts^6^. Errors such as these can have significant outcomes in the healthcare field, emphasizing the need for regulations around algorithm development.

A thorough assessment of collected data is crucial to confirm its representativeness of the target population and alignment with the intended use and setting. The data should be sufficiently large to support generalizability, and all data processing steps should be documented for implementing systems. The tool’s structure, UI design, and software should be clearly outlined for evaluation committees to ensure workflow compatibility.

Regulatory bodies have recognized that the historical data serving as the foundation for AI model training have the potential to produce models that perpetuate bias present in routine clinical care. For example, the EU Artificial Intelligence Act mandates high-quality data for training, denotes the risk of bias through proxy variables, and suggests various strategies for mitigating bias in AI systems. Additionally, in response to feedback from their AI regulation plan, the FDA acknowledged the risk of bias in historical data and presented the following strategies: regulatory science efforts, emphasis on diverse patient populations, real-world performance monitoring, and transparency requirements for manufacturers amongst other methods^4^. A number of tools exist to assess and correct for bias present in training data or models, including AI Fairness 360^32^, Google Fairness Indicators^33^, and Aequitas^34^.

### Model evaluation and validation and external algorithm evaluation

AI/ML models are characterized by the potential to “overfit” on training data, identifying nonbiological patterns that may predict the outcome of interest, and thus validation in independent populations is especially important. For example, in one commonly used public medical imaging dataset, 80% of cases with a pneumothorax had chest tubes in place. Models trained on this dataset may predict pneumothorax based on the presence of external devices (rather than the presence of a pneumothorax), leading to false positive predictions for pneumothorax in a patient with an ECMO cannula. Nonetheless, many approved devices are not externally validated.

External algorithm evaluation, selection, and model evaluation and validation were among the least discussed aspects in the reviewed literature, often with overlapping requirements. Despite this limited coverage, several key recommendations emerged. The primary suggestion across multiple sources was for external and internal validation dedicated to ensuring data representativeness and aligning quality and use with the intended application. These external validators could include governing bodies or independent expert committees, providing an additional layer of scrutiny beyond the development team.

Hond et al., Bedoya et al. (ABCDS framework), and Coombs et al. emphasized the importance of using either retrospective or prospective data for validation and generalizability assessment^8,11,14^. Overall, while specific methodologies varied, there was a consensus on the need for transparency in the evaluation process. For example, detailed documentation of evaluation criteria, methodologies, and results, making this information accessible to potential users, and such information will be required by regulatory bodies^7^.

### Deployment and integration

Deployment of AI tools in healthcare settings primarily focused on three critical areas: (i) shadow deployments, (ii) preemptive risk identification measures, and (iii) comprehensive end-user training.

To assess tool applicability and mitigate potential widespread consequences from errors, many sources recommended implementing a shadow deployment phase. During this period, health systems can evaluate risks, errors, and biases prior to full integration of the AI tool. This approach allows for real-world testing without compromising patient care, providing valuable insights into the tool’s performance in specific clinical contexts. Shadow deployments enable comparison of AI model performance with current operational standards and clinical outcomes, serving as a baseline for subsequent clinical evaluation protocols. Lastly, many sources recommended conducting a silent evaluation test or effectiveness assessment prior to deployment, emphasizing thorough testing in real-world settings before full implementation.

Ensuring proper AI system functionality and user acceptance requires a thorough understanding of workflow integration and potential failure modes. To effectively track these issues, healthcare institutions need to implement systems for identifying potential risk sources and establish clear channels for end-users to report errors. Some researchers suggest creating dedicated AI oversight committees to monitor and respond to these reports in real time. Care needs to be taken to ensure user interaction with AI tools remains meaningful and does not contribute to “alert fatigue.”

A major area of concern and guidance was the education of end-users. There is a risk of both over-reliance on automated systems, which can lead to increased errors, as well as under-reliance, when end-users do not trust model outputs. To mitigate this, end-users should receive comprehensive education on the AI tool’s objectives, expected outcomes, and potential limitations or biases. This training should also address the users’ own biases, which may influence tool usage. Model interpretability can also prevent inappropriate reliance on AI models—in one study, when AI visually outlined suspicious regions on chest radiographs, radiologists were less likely to accept incorrect AI classifications.

### Monitoring and maintenance

Frameworks largely emphasized the necessity of real-time monitoring for rapidly addressing major errors and implementing systems to audit and test bias, accuracy, and predictability. This ongoing assessment is essential for maintaining alignment between the AI system’s performance and the evolving needs of the target population.

Continuous assessment plays a vital role in confirming that the training data remains aligned with the characteristics of the target population. This process can effectively flag dataset shifts, which may occur when the distribution of training data significantly diverges from deployment data. Such shifts can manifest as changes in the distribution of input features, alterations in the relationship between input features and target variables, or modifications to the target variable itself. AI tools that learn from their outputs through feedback loops must be closely monitored, as unchecked adjustments can amplify errors or biases over time^14,19^. Moreover, AI tools capable of learning from their own output feedback loops require scrutiny^14^. Real-time monitoring provides a mechanism to assess whether these self-adjustments are valid or potentially contributing to errors. Most currently approved medical devices are based on ‘locked’ algorithms, where outputs do not change with continued use, but regulatory bodies will facilitate the assessment of more flexible models. For example, the FDA AI/ML framework requires specification of an algorithm change protocol (ACP) to define the key aspects of model retraining and performance evaluation for such continuously updating models^4^.

The dynamic nature of healthcare environments necessitates adaptations and updates to AI tools. Real-time monitoring serves as an early warning system, flagging when changes should be considered. However, implementing changes cautiously is crucial to avoid exacerbating risks. Additionally, continuous monitoring for data quality and model performance, with predefined thresholds for model retraining, ensures sustained effectiveness and adaptability of AI tools over time.

Furthermore, real-time monitoring facilitates the detection of potential biases that may emerge during deployment. By continuously analyzing the AI tool’s outputs across diverse patient populations, healthcare providers can identify and address any disparities in performance or recommendations that could lead to inequitable care. This ongoing evaluation is crucial for ensuring that AI tools maintain fairness and effectiveness across all demographic groups. Additionally, regular audits of tool outputs and user interactions can identify any potential bias by the end user.

## Discussion

A common challenge identified across articles was balancing actionable recommendations with guidance applicable across diverse AI applications. For example, the Collins et al. (TRIPOD + AI)^12,35^ checklist stood out for its comprehensive recommendations; however, they primarily focused on clinical prediction models and may not extend to newer generative AI applications, such as large language models in healthcare. This highlights the need for adaptable governance structures that can evolve standards and processes to address emerging technologies.

Given the combined guidance and recommendations across the included frameworks, we propose a HAIRA to provide actionable targets while addressing resource disparities (Table 4). This five-level maturity model recognizes that healthcare organizations vary significantly in their resources, expertise, and AI implementation needs. Drawing on the concepts of maturity seen in established frameworks like HIMSS’s digital transformation models, HAIRA provides a structured pathway for healthcare organizations to assess and advance their AI governance capabilities. The model spans from Level 1 (Initial/Ad Hoc), suitable for small practices beginning to explore AI implementation, to Level 5 (Optimized), appropriate for leading academic health systems pioneering industry standards. Each level addresses the seven domains identified in the literature: organizational structure, problem formulation, external algorithm evaluation, algorithm development, model evaluation, deployment integration, and monitoring maintenance. Supplemental Table 2 provides a summary version of this information. By providing clear benchmarks across these domains, HAIRA enables healthcare organizations to set realistic governance goals aligned with their resources and clinical needs, while establishing clear direction for improvement.

**Table 4:** Healthcare AI governance readiness assessment (HAIRA)

Readiness level	Summary characteristics	Breakdown	Required expertise/infrastructure
Level 1: Initial/Ad Hoc Basic awareness of AI governance needs with minimally structured processes. Healthcare systems that have not proactively defined an AI governance strategy but are exploring implementations begin here. Focus is on the safe adoption of validated commercial AI solutions with clear regulatory approval	Reactive approach to AI governance Heavy reliance on vendor expertise and support Limited internal capabilities for evaluation Focus on basic safety and compliance Minimal integration with existing systems	*Organizational structure*: no formal AI governance structure; decisions made by existing leadership. *Problem formulation*: basic identification of clinical needs without a formal evaluation process. *External algorithm evaluation*: relies on vendor claims and certifications. *Algorithm development*: no internal development; uses only validated commercial solutions. *Model evaluation*: confirmatory/acceptance testing of vendor solutions. *Deployment and integration*: manual processes with minimal integration. *Monitoring and maintenance*: reactive monitoring based on user reports	Basic IT infrastructure Clinical staff with general technology literacy External vendor support for AI tools Basic regulatory compliance capabilities
Level 2: Defined Emerging structured approach to AI governance with basic processes and oversight. Emphasis on structured evaluation and basic monitoring of AI solutions. Ideal minimum for systems with deployed AI	Basic documented processes for AI adoption Emerging capabilities to evaluate vendor claims Initial integration with clinical workflows Basic risk management procedures Structured approach to user training	*Organizational structure*: basic AI oversight committee with clinical and IT representation *Problem formulation*: structured needs assessment process with basic ROI evaluation *External algorithm evaluation*: documented evaluation criteria for vendor selection *Algorithm development*: limited customization of commercial solutions *Model evaluation*: structured testing protocols with basic validation processes *Deployment and integration*: basic integration planning with workflow consideration *Monitoring and maintenance*: regular performance reviews with basic metrics	Dedicated IT department Clinical informatics capabilities Basic data infrastructure Quality improvement team Contract management capabilities
Level 3: Established Comprehensive AI governance framework with standardized processes. Target for community and regional health systems. Capable of meaningful customization and validation of AI solutions	Standardized processes across the organization Ability to conduct independent validation Comprehensive risk assessment Integration with quality improvement Structured change management	*Organizational structure*: multi-disciplinary AI governance committee with defined roles and responsibilities *Problem formulation*: a comprehensive evaluation framework including clinical, technical, and ethical considerations *External algorithm evaluation*: robust validation process with internal testing capabilities *Algorithm development*: basic internal development capabilities with vendor partnerships *Model evaluation*: comprehensive validation process including bias assessment *Deployment and integration*: structured implementation process with change management *Monitoring and maintenance*: proactive monitoring with defined metrics and intervention thresholds	Data science team Clinical informatics team Enterprise data warehouse Dedicated staff with a focus on AI deployment Research capabilities Training infrastructure
Level 4: Advanced Sophisticated AI governance with robust processes and innovation capabilities. Target for major academic medical centers and leading healthcare systems. Capable of significant internal development and advanced validation	Proactive governance approach Advanced risk management Significant internal development capability Robust evaluation frameworks Innovation in deployment methods	*Organizational structure*: executive-level AI officer to lead strategy and governance with specialized subcommittees *Problem formulation*: strategic AI roadmap aligned with organizational objectives *External algorithm evaluation*: advanced testing capabilities with real-world validation *Algorithm development*: substantial internal development with research capabilities *Model evaluation*: comprehensive evaluation, including prospective studies *Deployment and integration*: seamless integration with existing workflows *Monitoring and maintenance*: real-time monitoring with automated alerts and interventions	Advanced data science and AI research teams Dedicated AI ethics committee Advanced computing infrastructure Comprehensive data governance Internal AI development capabilities Research partnerships
Level 5: Leading Leading-edge AI governance with continuous innovation and industry leadership. Aspirational level for top academic health systems and research institutions. Capable of setting industry standards and pioneering new approaches.	Industry-leading governance practices Novel AI applications development Setting evaluation standards Predictive risk management Continuous innovation in all areas	*Organizational structure*: center of excellence for AI governance with influence on industry standards *Problem formulation*: pioneering new AI applications with novel use cases *External algorithm evaluation*: setting industry standards for evaluation *Algorithm development*: leading-edge AI development with novel approaches *Model evaluation*: advanced validation methods, including multi-center studies *Deployment and integration*: innovative deployment models with continuous optimization *Monitoring and maintenance*: predictive monitoring with advanced analytics	World-class AI research capabilities Advanced computational resources Industry-leading data infrastructure Avenues to disseminate / translate innovation (e.g., incubator) Dedicated AI governance institute

Placement uses a minimum-domain (weakest-link) rule: failing any domain’s minimum criteria caps the overall level.

The proposed system emphasizes adaptability, recognizing that not all healthcare systems have equal access to advanced infrastructure or specialized personnel. For example, lower-resource settings may focus initially on foundational steps such as data standardization and basic validation of vendor claims, while higher-resource systems can engage in more complex tasks like multimodal data integration or real-time monitoring.

Self-placement proceeds domain-by-domain using a present or absent rubric across the seven domains starting with Level 1. We apply a minimum-domain (“weakest-link”) rule: the HAIRA level is the highest level for which every domain meets the level’s minimum standard; if any domain falls short, overall placement is capped at the next lower level. HAIRA can be scored at the health-system or service-line level; heterogeneity is expected (e.g., Radiology at Level 4 within a Level 2 system), and reporting both a unit- and system-level placement can guide staged scale-up.

For example, to qualify for Level 3 (production deployment), a site must have: (i) a named governance body with decision rights; (ii) documented internal validation including subgroup/fairness checks; and (iii) live monitoring with an incident-reporting pathway. If monitoring is absent, the site remains Level 2 even if other domains appear mature. To qualify for Level 4 (customization/development), the site must meet all Level 3 requirements and have defined software/data practices and strengthened change-management to support customization: lacking these caps the site at Level 3.

Rows represent interdependent bundles: capability in one domain presupposes a floor in adjacent domains. The minimum-domain rule makes this explicit, the lowest-scoring domain determines overall maturity. This weakest-link structure aligns with safety-critical operations, where a single missing control (e.g., monitoring or escalation) can undermine otherwise strong capabilities.

The findings from this literature informed the establishment of the level-based system introduced in HAIRA. Each organizational tier engages with every stage of the AI lifecycle, with specific actions determined by the resources available to that system. The required areas of expertise directly correspond to the tasks to be completed, thereby identifying the individuals and skill sets essential for achieving these objectives. Systems in the first levels have personnel limitations, so objectives have been modified to address this limitation.

A significant strength of this framework lies in its practicality: organizations can assess their current operational status using the readiness levels and corresponding expertise requirements previously defined and then compare these benchmarks with their in-house capabilities. The primary aim of this tool is to support system-wide oversight, enabling a central coordinating body to monitor AI deployment across all departments within a healthcare organization.

It is important to recognize that certain specialties, such as radiology, pathology, and dermatology, may have progressed further in their adoption of AI due to earlier and more extensive exposure to domain-specific tools. In these instances, such departments may advance to higher readiness levels and undertake more comprehensive assessments than their counterparts in other areas. Moreover, these leading departments can play a pivotal role in fostering system-wide advancement by sharing best practices and facilitating capacity-building. Ideally, all departments would possess the requisite expertise to conduct thorough analyses and ongoing monitoring of implemented AI tools. Achieving this would promote consistency, safety, and effectiveness in the integration of AI technologies across the healthcare enterprise.

Each level touches on each part of the AI lifecycle and outlines objectives to be addressed based on the resources that may be available to that system. The required expertise aligns with each goal and outlines the individual expertise necessary to achieve them.

Level 1: at level 1, organizations operate with basic awareness and minimally structured processes. Healthcare systems here have not proactively developed a formal AI governance strategy but are beginning to explore AI implementations. Their approach is primarily reactive, emphasizing the safe adoption of validated commercial AI solutions with clear regulatory approval. They rely heavily on vendor expertise and support, as they possess limited internal ability to evaluate AI tools. The main priorities are ensuring basic safety and compliance, with only minimal integration of AI into existing systems.

Key personnel at this stage include IT staff to maintain basic infrastructure and provide in-house expertise on understanding the AI tools, clinical staff who have general technology literacy, including EMR and the ability to comprehend the software being used, and external vendors who provide support for AI tools. There is also a need for individuals who can handle fundamental regulatory compliance tasks.

At this stage, the organization has no formal AI governance structure, with decisions managed by existing leadership. Clinical needs are identified in a basic way without a structured evaluation process. There is no internal algorithm development; instead, the organization exclusively adopts validated commercial AI solutions. Evaluation of these tools relies heavily on vendor claims, certifications, and limited confirmatory testing; these steps are often closely related and sometimes overlap in practice. Deployment and integration are manual, with minimal connection to existing systems, and monitoring is reactive, based on user reports.

External algorithm evaluation and model evaluation frequently converge, both focusing on accepting vendor assurances and conducting basic acceptance testing. They could be combined or more clearly distinguished, as their activities and recommendations often overlap in organizations at this level.

Level 2: at level 2, healthcare organizations begin implementing a formalized AI governance framework with defined processes and oversight to ensure responsible AI use. This includes establishing documented procedures for adopting AI technologies and developing the ability to critically assess vendor claims beyond simply accepting certifications. AI tools are progressively integrated into clinical workflows, supported by foundational risk management practices and a structured program to train users effectively. Governance is overseen by a committee composed of clinical and IT staff, ensuring cross-disciplinary input. Clinical needs are identified through a systematic assessment process that also considers the basic financial impact of AI projects. Vendor solutions may be customized to better fit organizational requirements, and models undergo standardized testing and validation to confirm performance. Deployment involves careful planning to align AI tools with existing workflows, while regular monitoring reviews key performance indicators to maintain safety and effectiveness.

Essential personnel include a dedicated IT team responsible for infrastructure and support, clinical informatics professionals who bridge technology and clinical practice, staff managing foundational data systems, quality improvement teams focused on outcomes, and contract managers handling vendor agreements. This level represents a foundational, structured approach essential for safe and effective AI deployment.

Level 3: at level 3, organizations implement a comprehensive AI governance framework with standardized processes applied consistently across the system, making this level suitable for community and regional health networks. They possess the capability to independently validate AI solutions, including conducting thorough risk assessments and integrating AI oversight into broader quality improvement initiatives. Change management is formalized to support the smooth adoption and adaptation of AI technologies. Governance is managed by a multidisciplinary committee with clearly defined roles and responsibilities, ensuring diverse expertise guides decisions. The evaluation of clinical needs incorporates clinical, technical, and ethical factors through a robust framework. Vendor algorithms undergo rigorous internal testing, while limited internal AI development occurs alongside vendor collaborations. Model validation is thorough, addressing performance as well as potential biases. Deployment involves a structured process with active change management, and monitoring is proactive, using defined metrics with established thresholds for intervention.

Key personnel include specialized data science and clinical informatics teams, supported by an enterprise data warehouse for centralized information management. Dedicated staff focus on AI deployment, backed by research capabilities and an established training infrastructure to continuously build organizational expertise. This level reflects a mature, well-integrated approach to AI governance and implementation.

Level 4: at Level 4, healthcare organizations, typically major academic medical centers and leading systems, maintain a sophisticated AI governance framework characterized by robust processes and strong innovation capabilities. Governance here is proactive, supported by advanced risk management and significant internal AI development, including substantial research activities. Evaluation frameworks are comprehensive, incorporating advanced testing methods and real-world validation, often through prospective studies. An executive-level AI officer leads the strategy and governance, supported by specialized subcommittees. AI initiatives align with a strategic roadmap integrated into organizational goals. Deployment is seamless within clinical workflows, while monitoring is real-time, utilizing automated alerts for timely interventions. Core resources include advanced data science and AI research teams, dedicated AI ethics committees, high-performance computing infrastructure, comprehensive data governance, strong internal development capabilities, and active research partnerships.

Level 5: at Level 5, top academic health systems and research institutions exemplify cutting-edge AI governance characterized by innovation and leadership that shape industry standards. These organizations develop groundbreaking AI applications and pioneer innovative use cases, setting the benchmark for evaluation criteria and risk management practices that incorporate predictive analytics. Governance is centered in a dedicated center of excellence, which also influences wider industry policies. AI development employs novel methods, supported by advanced validation techniques such as multi-center clinical studies to ensure robust performance and safety. Deployment models emphasize continuous optimization, adapting dynamically to clinical needs, while monitoring uses predictive analytics to anticipate and address risks proactively.

Resources at this level include leading AI research teams, state-of-the-art computational infrastructure, and leading data ecosystems. They also provide platforms for translating innovation into practice, such as incubators, and maintain dedicated AI governance institutes. Evaluation approaches go beyond traditional metrics, incorporating rigorous methodologies like A/B testing and sophisticated usability analyses, including mouse, cursor, and eye-tracking, to generate concrete evidence of AI tool effectiveness. This level represents the forefront of responsible, innovative AI integration in healthcare.

While resource availability is a critical factor in implementing AI governance, several other dimensions must also be considered. The demographic composition of the patient population significantly influences AI strategies, as diverse populations have distinct needs compared to homogeneous ones. Additionally, varying regulatory compliance and data privacy requirements necessitate customized governance approaches. Healthcare organizations should clearly define their objectives, as these goals inform the most suitable governance structures. For example, a system focused on enhancing diagnostic accuracy may require different oversight than one prioritizing operational efficiency. Finally, the existing organizational culture and established protocols play a crucial role in shaping AI governance implementation. Consensus across stakeholders, perspectives on the model being implemented, and personnel limitations can cause barriers to successful implementation^36^.

This work identified both emerging and consistent themes of healthcare AI governance as well as significant discrepancies between frameworks. Many of the recommendations identified in individual articles highlight critical elements that should be considered by healthcare systems, underscoring the need for comprehensive and unified frameworks. However, the few frameworks that are comprehensive require substantial personnel, expertise, and resource factors that may not be accessible to all healthcare systems.

In response to a need for actionable guidance that considers health system resources, we presented a new AI governance readiness model (HAIRA) so that healthcare organizations can assess and advance their AI governance capabilities based on expert recommendations outlined in this review. Integrating this tiered system into existing implementation frameworks could help bridge gaps identified in current models. For instance, frameworks like SALIENT and OPTICA provide detailed guidance on specific stages of AI implementation but often assume access to advanced resources or expertise^20,29^. Many regional and community health systems do not have in-house data science capabilities but instead may have data analysts supporting traditional quality improvement and reporting activities. Health systems may even outsource substantial portions of their IT needs, including using a hosted EMR system. Most of the proposed governance frameworks would be exceedingly difficult to implement in these settings. By incorporating a tiered structure, these frameworks could offer actionable pathways for systems at varying levels of readiness, ensuring broader applicability.

To develop appropriate organizational structures for various resource systems, we drew upon published governance models and framework proposals, with particular attention to the roles of key stakeholders as well as to the necessary institutional scaffolding, such as the formation of a central coordinating body. Healthcare presents unique organizational challenges, given the multitude of stakeholders involved, whose composition varies according to the context and scale of the healthcare system. Major actors include those responsible for financing, strategic planning, innovation, and the actual delivery of care^37^.

A study on the landscape of stakeholders involved in predicative analytics program in large healthcare systems indicated that while the majority have data scientists, clinical analytic experts, and physicians involved, only half had clinical informatics experts and information technology experts. Few institutions have clinical operation leaders and process improvement experts. These gaps were taken into account when assessing the expertise and skills available at each level of a system^38^.

To further inform our approach, we reviewed organizational and management literature addressing current theories of AI regulation in sectors outside medicine and considered how these regulatory strategies could be adapted to the medical context. This analysis allowed us to identify the resources and oversight mechanisms required at different organizational levels. A consistent finding across domains was the necessity of a central entity to ensure transparency and alignment throughout the lifecycle of AI implementation^39–41^.

We also considered the role of external third-party AI providers, particularly for systems without substantial in-house expertise, which may rely on buying tools^38^. In these contexts, it has been recommended that a central body coordinate with local teams, especially clinicians and patient representatives, who should have input into deployment decisions to maximize relevance and acceptability^42^. They can, in turn, work with third-party providers who can provide advanced technical expertise. However, it is crucial to remain vigilant about potential conflicts of interest inherent to for-profit organizations^43^. Maintaining an appropriate balance between engagement of these stakeholders and ensuring unbiased, proper use of AI tools is therefore essential.

Additionally, effective organizational approaches should include the launch of pilot projects with early clinician engagement, transparent communication, and the establishment of monitoring and feedback loops. Moreover, persistent challenges such as poor interoperability with legacy systems must be addressed to facilitate successful implementation^44^.

In conclusion, this synthesis focuses on comprehensive, multi-domain governance guidance; single-aspect guidance (e.g., ethics-only) and model-specific implementation reports were excluded by design. While this increases coherence, it may omit deep treatments of individual sub-domains. We partially mitigated this by expanding the search scope and drawing on organizational literature; however, some relevant cross-disciplinary sources may remain outside our biomedical database strategy. While we augmented biomedical sources with targeted management literature, a full cross-disciplinary systematic search of management databases was beyond scope and is a direction for future syntheses.

While not formally described in the frameworks identified through the search, a recent trend has been the creation of Chief AI Officer or similar roles^45^. This trend mirrors the emergence of Chief Medical Informatics Officers in recent years, reflecting the growing need for specialized technical expertise in healthcare infrastructure. As AI becomes increasingly integrated into clinical practice, healthcare organizations may need to adapt their leadership structures to empower an executive to dedicate a large portion or all of their effort toward developing the organization’s AI strategy and coordinating with governance committees to ensure judicious action.

A significant challenge in evaluating AI governance frameworks in healthcare is the limited empirical evidence of their effectiveness. Many of these frameworks are relatively new, and their implementation has not been extensively tested or published. This lack of data makes it difficult to determine whether these governance structures lead to better outcomes or achieve their initial goals of mitigating risks and maintaining tool excellence. To address this gap, we recommend conducting randomized controlled trials (RCTs) to assess various frameworks and recommendations. These “rapid” and electronically driven RCTs have been shown to be feasible and could help identify which guidelines effectively support their intended objectives. Designing and executing such trials presents considerable challenges, given the complexity of healthcare environments and the rapid evolution of AI technologies, but it will be critical to accurately allocate and justify investments in clinical AI. Future research should focus on developing methodologies that go beyond retrospective analyses and instead conduct prospective randomized evaluations.

Ultimately, the development of adaptable frameworks tailored to different levels of healthcare system capability is critical for equitable AI adoption. Such frameworks would not only address the current gaps but also provide clear guidance for systems aiming to advance their technological capacity over time. This article contributes to a more robust understanding of the current state and future directions of AI regulation in healthcare.

## Methods

We followed the PRISMA extension for systemic review guidelines for this systematic review on existing frameworks, guidelines, and checklists targeted towards AI implementation in the healthcare setting.

### Eligibility criteria

Primary eligibility criteria for inclusion in this systematic review were the presence of a holistic checklist, framework, or governance structure specifically addressing the implementation of AI in the healthcare setting. We intentionally broadened our criteria to encompass multiple types of guidance documents to ensure comprehensive coverage of literature providing actionable recommendations for evaluating AI in clinical practice. Evaluating the successful implementation of AI involves multiple dimensions, including bias, data quality, and outcome assessment, to name a few. Although extensive research has examined these elements individually, true success lies in addressing them collectively. Therefore, our review focuses on studies that propose comprehensive frameworks for AI implementation rather than those that target isolated components. Furthermore, we focused exclusively on healthcare settings due to the unique challenges posed by AI in this domain, including heightened concerns regarding patient privacy and the potentially life-threatening consequences of algorithmic errors, which are less salient in other sectors. While AI evaluation models exist for non-healthcare applications, these were considered out of scope for this review. We excluded articles outside healthcare; items that do not provide implementation guidance; single-aspect papers (e.g., ethics-only, deployment-only, or monitoring-only guidance); single-model case reports or performance evaluations without governance content; and non-peer-reviewed sources (conference abstracts, theses, book chapters, white papers, and other “grey” literature). A limited exception for grey literature was made in the case of sector-shaping organizational guidance (e.g., CHAI, NIST). These items were appraised with AACODS^46^ and labeled “grey” literature^47^. Notably, two prominent frameworks—FURM and OPTICA—were not captured in the initial systematic search due to indexing latencies in the primary databases for their respective journals. Given their significance within the field, these frameworks were manually included to ensure comprehensiveness of this review, as recommended by expert consensus^29,48^. Cross-disciplinary materials (e.g., general AI risk frameworks and organization/management concepts) were used narratively in the Discussion to contextualize governance role design and coordination mechanisms; they were not part of the systematic evidence base. This focus on comprehensive frameworks avoids fragmenting the synthesis into topic-specific best practices and supports coherent, system-level recommendations. Recognizing the rapid maturation of AI tools in healthcare, peer reviewed journal papers were included if they were published between 2019-2024, ensuring the relevance and applicability of the regulatory frameworks examined.

### Information sources, search strategy, and selection criteria

To identify relevant literature, we conducted a systematic search of PubMed/Medline for articles published in English from 2019 to 2024. The search strategy was developed collaboratively by a multidisciplinary team with expertise in machine learning and AI applications in healthcare and was further refined in consultation with an experienced academic librarian. The final search string was as follows: (“Artificial Intelligence” [MeSH] OR “AI”) AND (“Delivery of Health Care” [MeSH]) AND (framework OR governance OR checklist OR guideline). These results were filtered for 2019–2024, and the search was conducted on April 13, 2025.

Articles were included if they focused on healthcare settings and presented a comprehensive framework, governance structure, or checklist for AI implementation. We excluded articles that did not pertain to healthcare, did not provide guidance on AI implementation, or addressed unrelated topics. Additionally, non-peer-reviewed publications, conference abstracts, book chapters, and other forms of “grey literature” were excluded from the review. This approach enhances the reliability of our findings and ensures that resources meeting established academic standards are considered, while also streamlining the review process and maintaining methodological feasibility.

### Data charting process, appraisal, and results synthesis

Articles identified through our database searches were initially screened for eligibility based on title and abstract, with reference to the inclusion and exclusion criteria previously outlined. Any uncertainties regarding the eligibility of a particular article were resolved through discussion among reviewers BBJ, AZ, and RH. Subsequently, the full text of each potentially relevant article was retrieved and independently assessed for inclusion, applying the same pre-defined eligibility criteria to ensure consistency and rigor in the selection process

Included articles were closely analyzed to assess the structure (framework vs. checklist vs. guidelines) of evaluation, areas of focus, and specific recommendations made. Recommendations made by each article were extracted and documented. Recommendations were organized into the following seven categories based on widely recognized processes involved in the development and implementation of AI governance^25–27^ (Table 1)

Given that the included studies primarily consisted of prospective articles introducing new frameworks for AI implementation in healthcare, often conceptual rather than empirical, there were no established risk of bias assessment tools appropriate for this type of literature. Traditional risk of bias tools developed for randomized or observational clinical studies were not applicable. Instead, the quality assessment was conducted qualitatively by evaluating comprehensiveness and evidence of the frameworks. For studies where it was unclear whether a framework was introduced, two reviewers independently discussed these cases to reach consensus on inclusion and categorization. This process ensured thorough consideration and minimized subjective judgment bias. No automation tools were employed for the assessment. This approach was necessary to maintain transparency and rigor in the absence of formal risk of bias tools for conceptual framework studies.

### Risk of bias/credibility appraisal

Because included studies are conceptual frameworks rather than empirical clinical studies, standard risk of bias tools were not applicable. We qualitatively assessed completeness and evidentiary support for peer-reviewed frameworks and used AACODS for grey literature.

### Derivation of the healthcare AI governance readiness assessment (HAIRA)

We inductively coded all included documents for governance recommendations and grouped codes into seven domains: (1) governance body; (2) problem formulation; (3) external evaluation; (4) development/customization; (5) internal validation and fairness; (6) deployment and change-management; (7) monitoring. Two reviewers independently coded and reconciled differences. We observed discrete step-ups in resources, scope, and decision rights that informed five maturity levels (L1–L5). We operationalized levels with a minimum-domain rule (no additive scoring): a setting’s placement is the highest level for which all domains meet that level’s minimum criteria; any failing domain caps the level.

## Supplementary information

Supplementary Information

## Data Availability

No new data were generated or analyzed during the course of this systematic review. Findings were extracted from a systematic search of PubMed/medline for articles published in English between 2019-2024. The final search string was as follows: “Artificial Intelligence” [MeSH] OR “AI”) AND (“Delivery of Health Care” [MeSH]) AND (framework OR governance OR checklist OR guideline). These results werefiltered for 2019-2024 and the search was conducted on April 13, 2025.
