# Supplementary material for: Advancing healthcare AI governance through a comprehensive maturity model based on systematic review
Source: NPJ Digit Med. 2026 Feb 11;9:236. doi: 10.1038/s41746-026-02418-7 (PMC13004926; doi:10.1038/s41746-026-02418-7)
Supplement: Supplementary file 1 — Supplementary Information [file 41746_2026_2418_MOESM1_ESM.docx]

**Supplemental Table 1:** An overview of the selected publications recommendations across the different categories of the AI cycle. These are the distilled guidelines which may miss some of the details included in each articles. Four articles were excluded from this table due to the extensive level of detail in each section (i.e., checklist with a multitude of items per section).

| **Paper** | **Organizational Structure** | **Problem Formulation** | **External Algorithm Evaluation & Selection** | **Algorithm Development & Model Training** | **Model Evaluation & Validation** | **Deployment & Integration** | **Monitoring & Maintenance** |
| --- | --- | --- | --- | --- | --- | --- | --- |
| **Abràmoff et al. (2023)** |  |  | Conduct thorough evaluations of data quality, including input variables and outcomes, providing clear justification for outcome data collection methods and addressing any quality concerns or labelling issues if relevant. | Evaluate the specific health conditions and care processes where AI will be applied to promote health equity  Analyze the intended deployment environment of the AI/ML system to identify and address potential biases.  Ensure diversity in the AI development team, incorporating varied perspectives, backgrounds, and expertise to minimize perpetuation of historical biases.  Assess whether the training and test datasets adequately represent the characteristics of the target patient population, intended use, and input measurements to maximize the AI's applicability. Apply this consideration to prospective data as well. | Verify that the clinical study sample adequately represents the target patient population's key demographics, including age, gender, sex, race, and ethnicity, with sufficient sample size. |  | Assess the computability of the tool with the workflow of the health system setting. |

| **Paper** | **Organizational Structure** | **Problem Formulation** | | **External Algorithm Evaluation & Selection** | | **Algorithm Development & Model Training** | | **Model Evaluation & Validation** | **Deployment & Integration** | **Monitoring & Maintenance** |
| --- | --- | --- | --- | --- | --- | --- | --- | --- | --- | --- |
| **Bedoya et al.** | Organize diverse teams with specific skills:  1. Development team: data scientist responsible for model development  2. Executive-level committee: provides institution-wide oversight and governance. Includes Chief Health Informatics Officer, data science expert, and subcommittee leads. Including the following subcommittees:  A. Evaluation: Data scientists and physician-scientist with expertise on model development and evaluation. Responsibilities: separately review AI/ML tool performance and characteristics, offer recommendations on model readiness for implementation, and offer guidance on designing clinical outcomes study for development teams B. Implementation and Monitoring: Consists of individuals from IT with expertise in workflow design and implementation; clinical operation leads with expertise in project deployment and management. Responsibilities: Support deployment of models into clinical workflow, guide best practices, and help in identifying operational resources. Lead all central monitoring of the tools.  C. Consists of experts with regulatory and legal backgrounds and the IRB. Responsibilities: review FDA regulations, guidance, and policies, and assess applicability to each tool. Utilize this information to guide Oversight Committee. |  |  | | The Evaluation Committee should review retrospective results and evaluate the plans for deploying the tool with an emphasis on successful implementation within the existing workflow and UI design.  Teams should consider a silent evaluation phase as well as an  effectiveness evaluation. | |  | |  |  |
| **Paper** | **Organizational Structure** | **Problem Formulation** | **External Algorithm Evaluation & Selection** | | **Algorithm Development & Model Training** | | **Model Evaluation & Validation** | | **Deployment & Integration** | **Monitoring & Maintenance** |
| **Chen et al.** | Create diverse team of stakeholders | Reflect on the objectives of the model |  | | In data collection evaluate for imbalanced dataset, reflect on underserved and under studied populations and evaluate for any outcome choice bias | |  | |  | **Conduct Audits to identify specific harms** |

| **Paper** | **Organizational Structure** | **Problem Formulation** | **External Algorithm Evaluation & Selection** | **Algorithm Development & Model Training** | **Model Evaluation & Validation** | **Deployment & Integration** | **Monitoring & Maintenance** |
| --- | --- | --- | --- | --- | --- | --- | --- |
| **Reddy et al.** | Data governance panel consisting of AI developers, patient and target group representatives, clinical experts, and people with relevant AI, ethical, and legal expertise.   Revise organizational protocols and standards to guarantee that patients are informed about their healthcare provider's use of AI-assisted tools, including an explanation of the technology's capabilities and constraints.   Healthcare organizations should consider establishing new or leveraging existing expert groups to evaluate how well AI models align with specific clinical or operational requirements. However, it's important to recognize that not all current panels may possess the necessary expertise for this task. To address this gap, it’s recommended to implement a standardized benchmarking system for assessing AI models in healthcare settings.  Form a multidisciplinary clinical governance committee comprising of clinicians, managers, patient representatives, and technical and ethical experts to evaluate the efficacy and effectiveness of the AI model while overseeing privacy, safety, quality, and ethical considerations. | Outline clear clinical objectives to be achieved by the application of AI tools. | Establish a governance panel to scrutinize AI training datasets, ensuring they are adequately diverse and comprehensive to produce the intended model outcomes. This oversight aims to validate that the AI's foundation is representative and robust enough to support its designated functions. | The use of public datasets to develop AI may aid in efforts to minimize privacy breaches. | Apply interpretable frameworks, such as explainable AI (XAI) to support the decision-making process enhance the decision-making process. | Develop methods for educating healthcare professionals about the basics of AI and the tools implemented.  Educate the patient community and public. | Implement regular audits to assess AI systems for bias, precision, predictive power, decision-making transparency, and clinical effectiveness, using TRIPOD guidelines as a reference framework. |

**Supplemental Table 2.** Summary version of the Healthcare AI governance Readiness Assessment (HAIRA).

| **Category** | **Level 1:**  **Initial / Ad Hoc** | **Level 2: Defined** | **Level 3:**  **Established** | **Level 4: Advanced** | **Level 5: Leading** |
| --- | --- | --- | --- | --- | --- |
| **General Definition** | Basic awareness with minimal structured processes | Emerging structured approach with basic processes | Comprehensive framework with standardized processes | Sophisticated governance with innovation capabilities | Leading-edge governance with continuous innovation |
| **Organizational Structure** | No formal structure; ad hoc decisions | Oversight committee | Multi-disciplinary committee with defined roles | Executive-level office (e.g., CHAIO) with specialized subcommittees | Center of excellence guiding industry standards |
| **Problem Formulation** | Basic needs identification | Structured needs assessment | Comprehensive evaluation framework | Strategic AI roadmap | Pioneering new AI applications |
| **External Algorithm Evaluation** | Assessing Vendor claims only | Basic evaluation criteria | Robust validation process | Advanced testing capabilities | Setting industry standards |
| **Algorithm Development** | Commercial / External solutions only | Limited customization | Basic internal development | Substantial internal development | Leading-edge development |
| **Model Evaluation** | Basic acceptance testing | Structured testing protocols | Comprehensive validation | Prospective studies | Multi-center studies |
| **Deployment & Integration** | Manual processes | Basic integration planning | Structured implementation | Seamless integration | Innovative deployment models |
| **Monitoring & Maintenance** | Reactive monitoring | Regular performance reviews | Proactive monitoring | Real-time monitoring | Predictive monitoring |
| **Target Organizations** |  | Community hospitals | Regional health systems | Major academic centers | Top research institutions |
| **Required Non-Clinical Expertise** | IT support | Dedicated IT and informatics | Data science team | Advanced AI teams | World-class AI capabilities |

PRISMA 2020 Checklist

| **Section and Topic** | **Item #** | **Checklist item** | **Location where item is reported, page #** |
| --- | --- | --- | --- |
| **TITLE** | | |  |
| Title | 1 | Identify the report as a systematic review. | Title, #1 |
| **ABSTRACT** | | |  |
| Abstract | 2 | See the PRISMA 2020 for Abstracts checklist. | Abstract, #1 |
| **INTRODUCTION** | | |  |
| Rationale | 3 | Describe the rationale for the review in the context of existing knowledge. | Introduction, #1 |
| Objectives | 4 | Provide an explicit statement of the objective(s) or question(s) the review addresses. | Introduction, #2 |
| **METHODS** | | |  |
| Eligibility criteria | 5 | Specify the inclusion and exclusion criteria for the review and how studies were grouped for the syntheses. | Methods: Eligibility Criteria, #3-4 |
| Information sources | 6 | Specify all databases, registers, websites, organisations, reference lists and other sources searched or consulted to identify studies. Specify the date when each source was last searched or consulted. | Methods: Information Sources, Search Strategy, and Selection Criteria #4 |
| Search strategy | 7 | Present the full search strategies for all databases, registers and websites, including any filters and limits used. | Methods: Information Sources, Search Strategy, and Selection Criteria and Data Charting Process, Appraisal and Results Synthesis, #4 |
| Selection process | 8 | Specify the methods used to decide whether a study met the inclusion criteria of the review, including how many reviewers screened each record and each report retrieved, whether they worked independently, and if applicable, details of automation tools used in the process. | Methods: Data Charting Process, Appraisal and Results Synthesis #4. Information regarding the inclusion criteria is included. Papers were reviewed by 2-3 reviewers after the initial filtering for relevance process. |
| Data collection process | 9 | Specify the methods used to collect data from reports, including how many reviewers collected data from each report, whether they worked independently, any processes for obtaining or confirming data from study investigators, and if applicable, details of automation tools used in the process. | Methods: Data Charting Process, Appraisal and Results Synthesis, #4-5. Papers were reviewed and discussed by at least 2 reviewers. |
| Data items | 10a | List and define all outcomes for which data were sought. Specify whether all results that were compatible with each outcome domain in each study were sought (e.g. for all measures, time points, analyses), and if not, the methods used to decide which results to collect. | Methods: Data Charting Process, Appraisal and Results Synthesis, #4-5. Of note thee were not specific outcomes rather each article with a framework, governance structure, or checklist was reviewed and recommendations extracted into specific categories and reported out. |
|  | 10b | List and define all other variables for which data were sought (e.g. participant and intervention characteristics, funding sources). Describe any assumptions made about any missing or unclear information. | NA. As noted above there were not specific variables evaluated but rather each article was reviewed in whole for its systematic suggestions for evaluation of AI implementation in healthcare. |
| Study risk of bias assessment | 11 | Specify the methods used to assess risk of bias in the included studies, including details of the tool(s) used, how many reviewers assessed each study and whether they worked independently, and if applicable, details of automation tools used in the process. | Methods: Data Charting Process, Appraisal and Results Synthesis, #5. |
| Effect measures | 12 | Specify for each outcome the effect measure(s) (e.g. risk ratio, mean difference) used in the synthesis or presentation of results. | NA. This section is not applicable because our review focused on conceptual AI implementation frameworks without quantitative outcomes, so no numerical effect measures like risk ratios or mean differences were used or synthesized. |
| Synthesis methods | 13a | Describe the processes used to decide which studies were eligible for each synthesis (e.g. tabulating the study intervention characteristics and comparing against the planned groups for each synthesis (item #5)). | Methods: Data Charting Process, Appraisal and Results Synthesis, #4-5 |
|  | 13b | Describe any methods required to prepare the data for presentation or synthesis, such as handling of missing summary statistics, or data conversions. | NA. This section is not applicable to our paper because our review did not involve quantitative data synthesis or require data preparation methods such as handling missing summary statistics or data conversions. Since we focused on conceptual frameworks without numerical outcomes, there was no need for data transformation or preparation for meta-analysis or presentation. |
|  | 13c | Describe any methods used to tabulate or visually display results of individual studies and syntheses. | Methods: Data Charting Process, Appraisal and Results Synthesis, #4-5 |
|  | 13d | Describe any methods used to synthesize results and provide a rationale for the choice(s). If meta-analysis was performed, describe the model(s), method(s) to identify the presence and extent of statistical heterogeneity, and software package(s) used. | Methods: Data Charting Process, Appraisal and Results Synthesis, #4-5 |
|  | 13e | Describe any methods used to explore possible causes of heterogeneity among study results (e.g. subgroup analysis, meta-regression). | NA. We did not perform quantitative synthesis or meta-analysis of study results; therefore, no statistical heterogeneity assessment methods such as subgroup analysis or meta-regression were required. Our review focused on conceptual frameworks, which do not yield numerical effect estimates or variability metrics among studies. Consequently, exploring heterogeneity among quantitative results was not relevant to our synthesis. Any variability in framework characteristics was considered narratively rather than statistically. |
|  | 13f | Describe any sensitivity analyses conducted to assess robustness of the synthesized results. | NA. Our review focused on conceptual frameworks without numerical outcome data, and therefore sensitivity analyses were not applicable to assess the robustness of synthesized findings. |
| Reporting bias assessment | 14 | Describe any methods used to assess risk of bias due to missing results in a synthesis (arising from reporting biases). | NA. Since the included studies were primarily conceptual frameworks without numerical outcome data or effect estimates, there was no scope to assess or address potential reporting biases linked to missing summary results. Therefore, no specific methods were used to evaluate bias from missing results in a synthesis. Any discussion of bias focused on qualitative evaluation of study characteristics rather than domain-specific assessments of selective outcome reporting. |
| Certainty assessment | 15 | Describe any methods used to assess certainty (or confidence) in the body of evidence for an outcome. | NA. This section is not applicable to our review because we did not assess certainty or confidence in outcomes using formal tools like GRADE, as our included studies were conceptual frameworks without empirical outcome data. E.g., several oof the included manuscripts were perspectives where this would not be feasible. Consequently, no formal methods were used to evaluate the overall certainty of the evidence, which is typically relevant for quantitative syntheses of intervention effects. Instead, the review focused on qualitative appraisal of the clarity and comprehensiveness of the frameworks rather than grading the confidence in measurable effects. This reflects the nature of the evidence base and the review’s conceptual focus. |
| **RESULTS** | | |  |
| Study selection | 16a | Describe the results of the search and selection process, from the number of records identified in the search to the number of studies included in the review, ideally using a flow diagram. | Results, 5-12 |
|  | 16b | Cite studies that might appear to meet the inclusion criteria, but which were excluded, and explain why they were excluded. | Results, 5-12 |
| Study characteristics | 17 | Cite each included study and present its characteristics. | Results, 5-12 |
| Risk of bias in studies | 18 | Present assessments of risk of bias for each included study. | This could not be done formally but we assessed specifically the fact that nearly all included papers come from major academic health systems and include this as a current limitation of the field in the discussion. |
| Results of individual studies | 19 | For all outcomes, present, for each study: (a) summary statistics for each group (where appropriate) and (b) an effect estimate and its precision (e.g. confidence/credible interval), ideally using structured tables or plots. | NA. As noted above this included conceptual frameworks and no numerical outcomes were evaluated. |
| Results of syntheses | 20a | For each synthesis, briefly summarise the characteristics and risk of bias among contributing studies. | NA. We did not perform quantitative synthesis of study results |
|  | 20b | Present results of all statistical syntheses conducted. If meta-analysis was done, present for each the summary estimate and its precision (e.g. confidence/credible interval) and measures of statistical heterogeneity. If comparing groups, describe the direction of the effect. | NA. We did not perform quantitative synthesis or meta-analysis of study results. |
|  | 20c | Present results of all investigations of possible causes of heterogeneity among study results. | NA. We did not perform quantitative synthesis or meta-analysis of study results; therefore, no statistical heterogeneity assessment methods were required. |
|  | 20d | Present results of all sensitivity analyses conducted to assess the robustness of the synthesized results. | NA. Our review focused on conceptual frameworks without numerical outcome data, and therefore sensitivity analyses were not applicable to assess the robustness of synthesized findings. |
| Reporting biases | 21 | Present assessments of risk of bias due to missing results (arising from reporting biases) for each synthesis assessed. | NA. Since the included studies were primarily conceptual frameworks without numerical outcome data or effect estimates, there was no scope to assess or address potential reporting biases linked to missing summary results. Therefore, no specific methods were used to evaluate bias from missing results in a synthesis. Any discussion of bias focused on qualitative evaluation of study characteristics rather than domain-specific assessments of selective outcome reporting. |
| Certainty of evidence | 22 | Present assessments of certainty (or confidence) in the body of evidence for each outcome assessed. | NA. This section is not applicable to our review because we did not assess certainty or confidence in outcomes using formal tools like GRADE, as our included studies were conceptual frameworks without empirical outcome data. Consequently, no formal methods were used to evaluate the overall certainty of the evidence, which is typically relevant for quantitative syntheses of intervention effects. Instead, the review focused on qualitative appraisal of the clarity and comprehensiveness of the frameworks rather than grading the confidence in measurable effects. This reflects the nature of the evidence base and the review’s conceptual focus. |
| **DISCUSSION** | | |  |
| Discussion | 23a | Provide a general interpretation of the results in the context of other evidence. | Discussion, 15-24 |
|  | 23b | Discuss any limitations of the evidence included in the review. | Discussion, 15-24 |
|  | 23c | Discuss any limitations of the review processes used. | Discussion, 15-24 |
|  | 23d | Discuss implications of the results for practice, policy, and future research. | Discussion, 15-24 |
| **OTHER INFORMATION** | | |  |
| Registration and protocol | 24a | Provide registration information for the review, including register name and registration number, or state that the review was not registered. | NA. This systematic review was not registered in a prospective register due to timing constraints; by the time the decision to conduct the review was finalized and the protocol developed, the opportunity for timely registration had passed. While registration is recommended to promote transparency and minimize bias, the rapidly evolving nature of the AI implementation frameworks field necessitated expeditious review completion. Despite this, the review was conducted rigorously following established guidelines to ensure transparency and reproducibility |
|  | 24b | Indicate where the review protocol can be accessed, or state that a protocol was not prepared. |  |
|  | 24c | Describe and explain any amendments to information provided at registration or in the protocol. |  |
| Support | 25 | Describe sources of financial or non-financial support for the review, and the role of the funders or sponsors in the review. | None. |
| Competing interests | 26 | Declare any competing interests of review authors. | No Disclosures. |
| Availability of data, code and other materials | 27 | Report which of the following are publicly available and where they can be found: template data collection forms; data extracted from included studies; data used for all analyses; analytic code; any other materials used in the review. |  |

*From:*  Page MJ, McKenzie JE, Bossuyt PM, Boutron I, Hoffmann TC, Mulrow CD, et al. The PRISMA 2020 statement: an updated guideline for reporting systematic reviews. BMJ 2021;372:n71. doi: 10.1136/bmj.n71. This work is licensed under CC BY 4.0. To view a copy of this license, visit <https://creativecommons.org/licenses/by/4.0/>
